# In-hospital outcome after trochanteric femur fractures is related to preoperative delay but not to the time of day of the procedure: a nationwide retrospective cohort study of 7184 patients

**DOI:** 10.1007/s00068-025-03012-4

**Published:** 2025-11-14

**Authors:** Valentina Egger, Anne Sophie Mittlmeier, Claudio Canal, Valentin Neuhaus

**Affiliations:** 1 University Clinic for Orthopedic Surgery and Traumatology, Inselspital Bern, Freiburgerstrasse 18, Bern, 3010 Switzerland; 2 Division of Trauma Surgery, University Hospital Zurich (USZ), University of Zurich (UZH), Raemistrasse 100, Zurich, 8091 Switzerland; 3 Department of Surgery, Cantonal Hospital Thurgau, Pfaffenholzstrasse 4, Frauenfeld, 8500 Switzerland

**Keywords:** Preoperative delay, Early surgery, Trochanteric femur fracture, Outcome, Case-control study

## Abstract

**Introduction:**

Hip fractures are a growing public health concern, and efficient management is critical. Surgical timing is a key modifiable factor influencing outcomes, but fracture-specific data – particularly for trochanteric fractures – remains limited. Moreover, concerns regarding the safety of nighttime surgery may contribute to procedural delays.

**Aim:**

This study investigates how time to surgery and timing of surgery (day vs. night) affect in-hospital complication and mortality rates in patients undergoing trochanteric fracture fixation.

**Materials and Methods:**

This nationwide registry study included 7,184 patients who underwent closed reduction and internal fixation for a trochanteric femur fracture. Patient demographics, surgical details, hospitalization characteristics, and discharge data were analyzed. Patients were stratified by preoperative interval (< 24 vs. >24 h) and surgery starting time (day vs. night). The primary outcome was in-hospital complications; secondary outcome was in-hospital mortality. Unpaired t-tests, chi-square tests, and backward stepwise binary logistic regression were performed (*p* < 0.05 considered significant).

**Results:**

Patients were predominantly elderly, chronically ill women with statutory insurance. The overall in-hospital complication rate was 17%, and the mortality rate was 3.4%. Surgery delayed beyond 24 h was associated with higher complication rates, longer operative times and hospital stays, and lower discharge mobility. Delay was an independent risk factor for complications and was associated with increased mortality, though not independently. No significant differences were observed between day and night surgeries.

**Discussion:**

Our fracture-specific findings align with current clinical guidelines: surgery within 24 h leads to better in-hospital outcomes. Delays were linked to a 38% rise in complications and a 74% increase in mortality. Nighttime surgery was demonstrated to be safe and should not be avoided without medical justification. The study’s limitations include its retrospective design and its focus on in-hospital outcomes only.

**Conclusion:**

Minimizing surgical delay for trochanteric fractures is fundamental. System-level improvements and interdisciplinary coordination are needed to ensure timely care. Given the aging population, the implementation of streamlined treatment pathways is increasingly important.

## Introduction

Hip fractures represent a major public health issue and are the most severe yet common consequence of osteoporosis [1–3]. They account for a substantial loss of healthy life years, driven by high rates of complications, mortality, long-term disability, and loss of autonomy [1, 2, 4–6]. The rising incidence of hip fractures due to demographic aging is already imposing significant costs on healthcare systems worldwide [3, 7]. Effective management of hip fractures is therefore a priority to reduce their burden on individuals, society and healthcare infrastructures.

While many risk factors are well known, yet largely non-modifiable [8, 9], timing of surgical intervention is a critical modifiable factor influencing patient outcomes. Timely surgery is essential to alleviate pain, enable early mobilization, and prevent complications, such as urinary tract infections (OR 0.57 [10], 0.44 [11]), pneumonia (OR 0.83 [12]), congestive heart failure (OR 0.77 [12]), delirium (0.47 [13, 14]), pressure ulcers (OR 0.55 [10]; 0.25 [11]), deep vein thrombosis (OR 0.61 [10]; OR 1.1/d [15]). Furthermore, prolonged bedrest leads to muscular and systemic deconditioning [16].

Conversely, delayed surgery is associated with longer hospital stays, higher complication rates, and increased mortality [10, 12, 17–35].

Current international guidelines recommend surgery within 24 to 48 h of hospital admission, with some advocating for even earlier intervention [21, 36–42]. However, the precise threshold for acceptable delay remains under debate, complicated by conflicting study results and logistical feasibility [10, 12, 20, 23, 26, 28, 43–48].

Importantly, evidence on the timing of surgery often overlooks distinctions between different hip fracture types - particularly trochanteric versus femoral neck fractures. These subtypes vary in anatomical location, treatment strategies, patient demographics, and clinical outcomes. Trochanteric fractures - being extracapsular – are associated with a higher primary blood loss than intracapsular femoral neck fractures [49]. They are usually managed with closed reduction and intramedullary nailing, a procedure that is relatively fast, resource-efficient, and technically less demanding than arthroplasty [50, 51]. In contrast, femoral neck fractures are intracapsular and commonly require internal fixation or hip arthroplasty. Patients with trochanteric fractures tend to be older, with lower baseline mobility and fitness, and have worse clinical outcomes including higher complication and mortality rates, as well as prolonged hospitalization and rehabilitation [34, 52–54]. Grouping both fracture types in research may obscure important fracture-specific insights – highlighting the need for more targeted research on the optimal timing of surgery, particularly for trochanteric fractures.

A further complication lies in the challenge of performing hip fracture surgery during nighttime hours. Despite the urgency of timely intervention, overnight procedures raise safety concerns due to surgeon fatigue, reduced staffing, and slower workflows [55]. These system-level barriers can undermine guideline adherence and make scheduling within the recommended timeframe difficult. Balancing the benefits of early surgery with the potential risks of nighttime operations remains a pressing issue, particularly as healthcare systems strive to optimize outcomes under constrained resources.

## Aim

This study investigates the influence of preoperative delay and nighttime surgery on in-hospital complication and mortality rates in patients with trochanteric fractures. We hypothesize that surgery performed within 24 h improves outcomes, irrespective of whether it is performed during day- or nighttime.

With the fracture-specific focus of this research, we seek to address the limitations of prior studies and contribute evidence regarding the optimal timing of surgery for trochanteric hip fractures. Our findings aim to guide clinical practice and improve outcome.

## Materials and methods

### Study design and setting

We analyzed the data of patients with trochanteric femur fractures from the prospective surgical registry of the working group for quality assurance in surgery (“Arbeitsgemeinschaft für Qualitätssicherung in der Chirurgie” or AQC). The AQC is a voluntary association of physicians in Switzerland formed with the aim of evaluating the in-hospital outcomes of surgical patients. Since almost 30 years 166 departments/attending doctors contributed 2.1 million anonymized cases with the purpose of ensuring surgical quality in Switzerland [56]. The database contains information about (a) the hospitalization and (b) the procedure(s). The diagnosis is coded according to the International Classification of Diseases, Tenth Edition (ICD-10) - a clinical cataloguing system developed by the World Health Organization (WHO) [57]. Procedures are coded according to the Swiss operation classification ‘CHOP’ [58]. Because only de-identified data from this national database was used, no institutional review board approval was required.

### Study subjects

The inclusion criteria for the current study consisted of patients with the main diagnosis trochanteric femur fractures (ICD code S72.1) and main surgery by closed reduction and internal fixation (CRIF, CHOP code 79.15). Under the considerations of ICD and CHOP 9094 patients were elicited, 1910 cases were excluded due to missing data, especially the starting time of the surgery to ensure accurate timing analysis, leaving a total of 7184 patients with operatively treated trochanteric femur fractures between January 5th, 2001, and April 19th, 2024.

### Variables and outcome measures

The following parameters from the AQC database were considered for this study: patient’s age, sex, American Society of Anaesthesiologists (ASA) physical status classification system score, type of insurance (statutory or private), surgeon class (senior attending, junior attending, resident), length of surgery in minutes, the occurrence of any complication (yes or no), length of hospital stay in days, and discharge status (home, rehabilitation clinic, nursing home, old people’s home, death, and others). Complication comprised general (e.g. pneumonia, renal dysfunction), intraoperative (e.g. iatrogenic fractures, tissue damage) or postoperative (e.g. bleeding, infection, malreduction). No standardized frailty scoring system was applied and the fractures were not subclassified according to severity.

We categorized the patients according to the preoperative interval (less or more than 24 h) to evaluate the influence of surgical delay.

For further analysis, we stratified the patients into two additional groups based on the time of surgery start: ‘DAY,’ for surgeries starting between 7:00 a.m. and 6:59 p.m., and ‘NIGHT,’ for surgeries starting between 7:00 p.m. and 6:59 am.

The occurrence of any complication during the hospitalization was the primary outcome, whereas in-hospital mortality was the secondary outcome.

### Statistical analysis

The data was downloaded from the AQC database using the AdjumedAnalyze software (Adjumed Services AG, Zurich, Switzerland). The statistical analysis was executed in SPSS version 29.0.2.0 (IBM Corp., Armonk, NY, USA). The chi-square test was employed for analysis of the categorical data, presented as absolute numbers of patients and their corresponding percentages. An unpaired t-test was performed to compare the numerical data, presented as means ± standard deviation (SD). A *p*-value of < 0.05 was considered to indicate statistical significance. We utilized backward stepwise binary logistic regression as a statistical tool to explore and identify independent risk factors associated with the likelihood of any complication and death. ASA V and ASA I were excluded as predictors for mortality, since they were always associated (or not at all) with death.

## Results

### Study population

A total of 7184 patients were included in the study. The mean age was 82 ± 11 years, the patients had predominantly a female sex (73%). An ASA score of II or III was recorded in 90% of patients, with 45% having ASA II and 45% ASA III, followed by ASA I with 6.3% and ASA IV with 2.9%. Statutory insurance covered 74% of the patients, and 42% of the surgeries were done by junior consultants, 30% by senior consultants, and 28% by residents. The length of surgery was 68 ± 39 min. The patients stayed 10 ± 6 days. After the hospital stay, 26% went home, 29% were discharged to a rehabilitation clinic, 15% to an old people’s home, and 19% to a nursing home (Table 1).

**Table 1:**
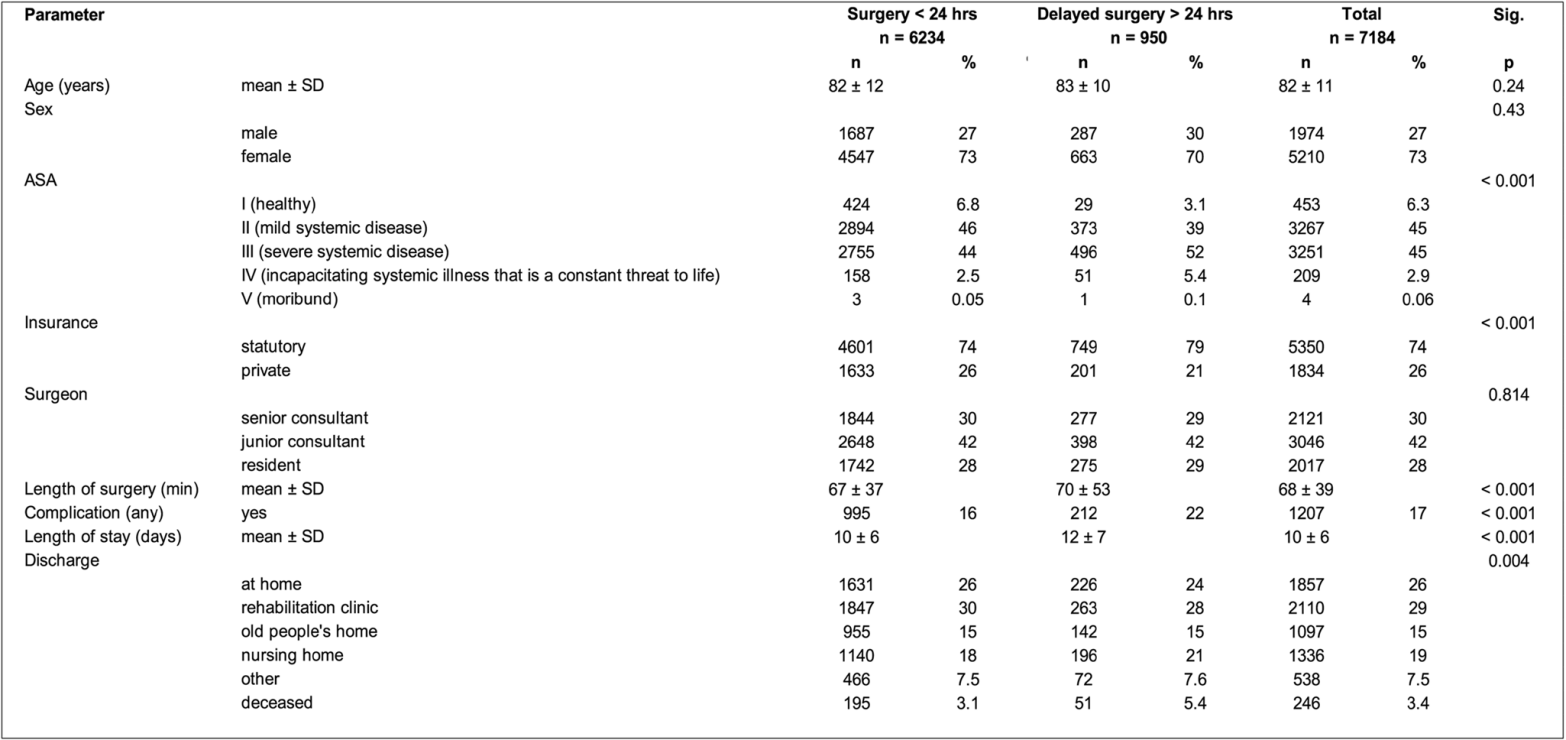
Patient and hospitalisation characteristics

*ASA* American Society of Anaesthesiologists classification system, *SD* Standard Deviation, *Sig.* Significance

### Preoperative delay of > 24 h

Nine hundred and fifty patients (13%) received surgery after more than 24 h of hospitalization. These patients had similar age and sex as the group with a delay of less than 24 h, however, they had a significantly higher ASA score and a marginally greater coverage under statutory insurance.

In the group with preoperative delay over 24 h, we observed a statistically significant difference in the length of surgery (70 ± 53 vs. 67 ± 37 min), occurrence of any complication (22% vs. 16%), and length of hospitalization (12 ± 7 vs. 10 ± 6 days). Also, patients were more often discharged to a nursing home (21% vs. 18%) or deceased (5.4% vs. 3.1%) and less often discharged home (24% vs. 26%) or to a rehabilitation clinic (28% vs. 30%) (Table 1).

### Complications

The overall complication rate was 17%. Surgery delayed beyond 24 h was associated with a 37.5% increase in the complication rate (22% vs. 16%). Binary logistic regression analysis identified delayed surgery > 24 h, an increased ASA score, male sex, higher age and longer surgery duration as significant risk factors for the occurrence of complications. (Table 2)

**Table 2:**
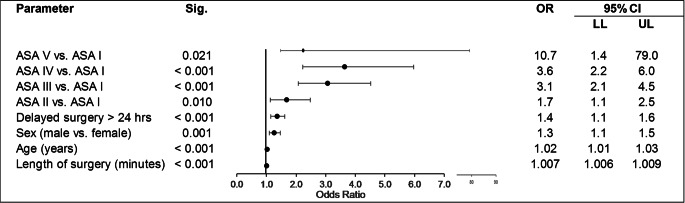
Predictors of complications

*CI* confidence intervall, *LL* lower limit, *OR* odds ratio, *Sig.* significance, *UL* upper limit

Hence, delayed surgery after 24 h was confirmed as an independent risk factor, indicating a 38% increased risk for the occurrence of any complication.

### Mortality

The overall in-hospital mortality was 3.4%. Delayed surgery was significantly associated with a 74% increase in the mortality rate (5.4% vs. 3.1%), but not a significant predictor for mortality in regression analysis. Binary logistic regression identified complications and advancing age as significant risk factors for mortality (Table 3).

**Table 3:**
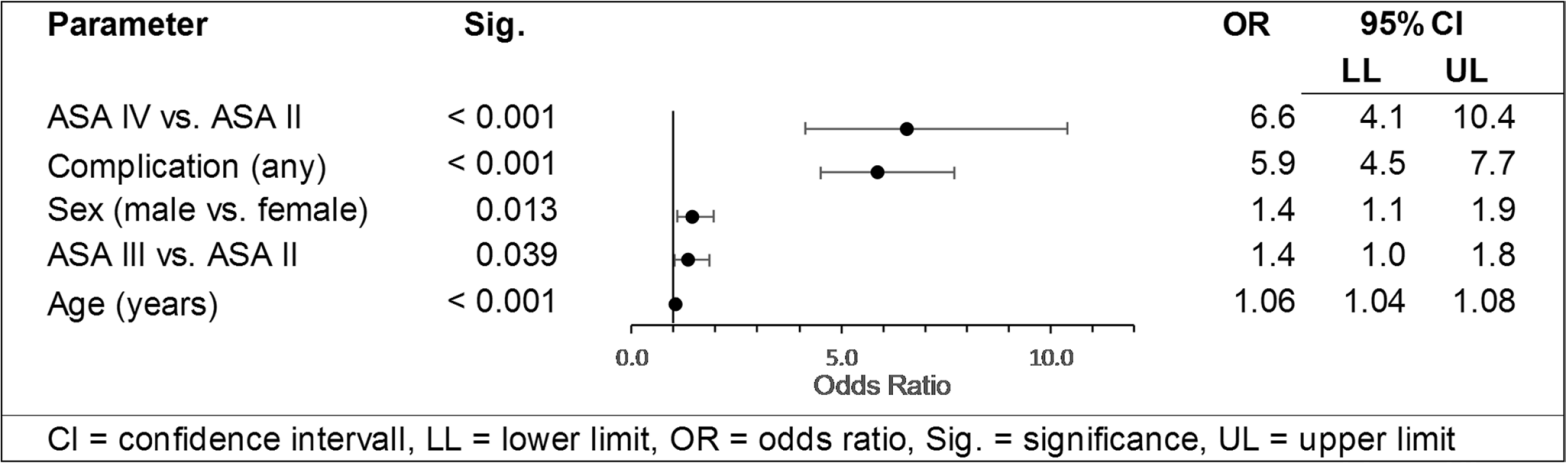
Predictors of mortality

*CI* confidence intervall, *LL* lower limit, *OR* odds ratio, *Sig.* significance, *UL* upper limit

### Daytime versus nighttime surgery

Among 2406 patients, the exact starting time of the surgery was available. The complication rate was similar between the daytime and nighttime groups (*p* = 0.263), and mortality rates were also not statistically different (*p* = 0.15). Including this dichotomous parameter in the logistic regression, the timing of surgery was not retained in the final regression model (Table 4).

**Table 4:**
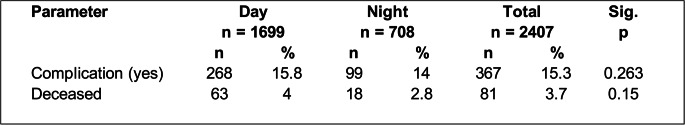
Daytime and nighttime surgery

## Discussion

### Key findings

Our study contributes additional evidence to the ongoing debate, whether delayed surgery > 24 h or nighttime surgery starting between 7:00 p.m. and 6:59 am increases the in-hospital complication and mortality rate. In this nationwide retrospective cohort study (evidence level III), we analysed 7184 patients with trochanteric femur fractures treated with CRIF during an almost 24-year period. In our study, patients receiving early surgery within 24 h of admission showed a better in-hospital outcome. Patients undergoing delayed surgery had an increased complication rate (22% vs. 17%). Preoperative delay was found to be an independent risk factor for the occurrence of complications, alongside with an increased ASA score, male sex, higher age, and longer surgery duration. Delayed surgery was significantly associated with mortality (5.4% vs. 3.1%), but not a significant predictor for mortality in the regression analysis. Independent risk factors for mortality were higher ASA scores, the occurrence of any complication, male sex, and advancing age. Nighttime surgery was not associated with a higher complication or mortality rate, nor was it a significant risk factor in multivariate analysis. While the timing of hip fracture surgery has been studied extensively, fracture-type-specific analyses remain rare.

### Implications

Our findings highlight the critical role of early surgery within 24 h of admission in reducing in-house complications, mortality and hospital stay, particularly for trochanteric femur fractures. Preoperative delay emerged as the most modifiable predictor of adverse outcomes. Surgical delay did not predict mortality independently, but the occurrence of any complication. Concerns about night-time surgery should not justify delays. Instead, strategic prioritization can optimize resource allocation without compromising patient safety. Achieving timely intervention requires addressing systemic delays—especially staffing and surgical capacity—through hospital-level improvements and policy support. Multidisciplinary coordination, designated trauma rooms, and orthogeriatric care have proven effective in streamlining processes and improving outcomes. Fracture-specific guidance for trochanteric fractures could enhance current hip fracture protocols. While implementing a < 24-hour surgery target poses challenges, doing so benefits both patients and healthcare systems.

### Known evidence

#### Demographics and baseline-outcome

The typical patient in our study was an elderly woman with chronic comorbidities, reflecting a 3:1 female-to-male ratio consistent with existing literature [7, 20, 26, 59–62]. Our in-hospital mortality rate for trochanteric femur fractures was 3.4%, slightly above the 2.6% national average for all hip fractures in Switzerland but well within the international range of 1.9-7.0.0% for trochanteric fractures [17, 18, 28, 46, 61–66]. Reported in-house complication rates globally vary between 12 and 46% [17, 46, 62–64, 66].

#### Early surgery benefits for patients with hip fractures generally

Multiple studies support early surgery (within 24 h) for patients with hip fractures, showing reduced 30-day and one-year mortality, especially in elderly patients without reversible contraindications. Welford et al.’s 2021 meta-analysis of 46 studies confirmed these findings (RR 0.86), even when comparing to a 24–36 h window [26]. Earlier meta-analyses reported significantly fewer complications with surgery within 24 h, yet significant mortality benefits appearing only at one-year postoperatively [10, 20]. Patients with comorbidities seem to benefit most of early surgery [20, 67]. The Hip Fracture Accelerated Surgical Treatment and Care Track (HIP ATTACK) RCT found no additional advantage of surgery within 6 h versus 24 h for complications or mortality but did show quicker mobilization and shorter hospital stay [33]. While medical optimization of patients with severe and reversible conditions is crucial and guidelines exist, surgical delays in comorbid patients were also shown to reflect capacity challenges [68, 69].

#### Comparison with other studies

Several studies support an association between surgical delays beyond 24 h and increased complication rates in trochanteric femur fractures. Daginnus et al. found this effect in Germany, but only for trochanteric – not femoral neck – fractures [34]. Tanaka et al. observed similar results in Japan, albeit assessing a 48-hour-threshold for surgery [19]. Vidan et al. reported the highest reported complication rate of 46% in acute hip surgery in Spain with a median delay of 72 h, largely due to operating room limitations [64]. Pincus et al., in a Canadian study of 42,000 patients, identified 24 h as a key threshold for increased complications and 30-day mortality, though without fracture type differentiation [28]. In a Swedish register analysis, Greve et al. linked delays over 24 h to worse outcomes, particularly in higher-risk patients [ASA III/IV and women] [12, 70]. Other studies, such those by Delaveau [29] and Schermann [31], reinforce mortality benefits for surgery within 24 h, especially in the elderly. However, some studies like those by Tanner [47], Orosz [48], and Saul [46] show mixed or minimal effects, likely due to small sample size, short delays, or heterogenous patient population.

### New contributions and strength of the study

Despite extensive research upon acute hip fracture, many studies fail to differentiate between femoral neck and trochanteric fractures - two distinct entities which differ in pathophysiology, treatment, and outcomes [34, 52, 53, 71, 72]. In trochanteric fractures, the lack of capsular confinement facilitates uncontained bleeding, soft tissue injury, inflammatory response, and are linked to higher complication and mortality rates, especially in older, frailer patients [73–77]. As the population ages, the incidence of trochanteric fractures is rising, particularly among men, making them increasingly relevant [71].

Our study focuses specially on trochanteric fractures, confirming the association between surgical delay beyond 24 h and worse in-hospital outcome, while nighttime surgery was not found to be a risk factor. Closed reduction and intramedullary nailing, the standard treatment, is minimally invasive, resource-efficient, and manageable even by junior surgeons [46, 78]. Avoiding night surgery due to staffing or logistical concerns contributes unnecessarily to surgical delay [6, 45, 52, 53, 62, 71, 72]. While perceptions of nighttime risk persist, evidence from our study and others does not support increased harm in timely off-hour hip operations [79–81]. The large, multi-center cohort of over 7,000 patients strengthens the generalizability of our findings and underscores the need for fracture-specific guidelines - particularly as demographic shifts point to a growing burden of trochanteric fractures in aging populations [7].

### Limitations

This study is limited trochanteric fractures treated with closed reduction and internal fixation; thus, findings cannot be generalized to complex cases or other procedures. Its retrospective design restricts causal inference, and stratifying solely by time to surgery may overlook necessary delay for medical optimization. Furthermore, procedural developments regarding surgical technique or management might have occurred over the 24 years observed. Registry-based analysis lacks detailed patient-level data, raising the risk of unmeasured confounding (e.g. reason for delay, hospital specific protocols, preoperative management). The AQC registry does not assess subdivisions of fracture or fixation type, neither orthogeriatric treatment, denying further analysis. No adjustments or propensity matching were performed. Generalizability is limited to healthcare systems with similar structures, and outcomes were restricted to the in-hospital period.

## Conclusion

Minimizing surgical delays in trochanteric fractures is crucial, as unwarranted postponement increases the risk for adverse outcomes (complications, mortality) and costs without proven benefit. Night-time surgery was not associated with higher perioperative risk. While short delays for medical optimization may be justified, organizational improvements and orthogeriatric co-management are essential to ensure timely, safe surgery. With the rising incidence of fragility fractures, focused research and early multidisciplinary care remains key to improving outcomes and healthcare efficiency.

## Data Availability

The research data can be accessed through the AQC database.

## Declarations

### Statement of location where the work was performed

Division of Trauma Surgery, University Hospital Zurich (USZ), University of Zurich (UZH), Raemistrasse 100, 8091 Zuerich, Switzerland
